# Condition Monitoring of In-Service DFIGs Working Under Non-Stationary Conditions via NsHOTA: A Motor Current Signature Approach

**DOI:** 10.3390/s25247451

**Published:** 2025-12-07

**Authors:** Sandra Delfa-Baena, Estefania Artigao, Carla Terron-Santiago, Andres Honrubia-Escribano, Jordi Burriel-Valencia, Emilio Gomez-Lazaro

**Affiliations:** 1 Institute for Energy Engineering, Universitat Politècnica de València, Camino de Vera s/n, 46022 Valencia, Spain; sandelba@etsii.upv.es (S.D.-B.);; 2 Renewable Energy Research Institute (IIER), University of Castilla-La Mancha, 02071 Albacete, Spainandres.honrubia@uclm.es (A.H.-E.);

**Keywords:** condition monitoring, current signature analysis, DFIG, HOTA, wind turbine, fault diagnosis, non-steady-state analysis, time degradation analysis

## Abstract

The reliability of wind turbines largely depends on the ability to detect electrical and mechanical faults under variable operating conditions. This paper applies the Non-steady-state Harmonic Order Tracking Analysis (NsHOTA) method to the diagnosis of doubly-fed induction generators (DFIGs) in real wind turbines. Unlike other steady-state and transient techniques, NsHota stabilizes and enhances fault components in any operating regime, allowing for more in-depth analysis. Therefore, this method enables highly accurate fault diagnosis, allowing the measurement and analysis of small degradations over time. The method is validated using eight months of field data from an 850 kW DFIG previously diagnosed with mixed eccentricity. The results demonstrate that NsHOTA improves the consistency and quality of fault feature extraction, reduces background noise, and avoids false negatives under steady and non-steady regimes. In the real data test, NsHOTA is also compared with the steady-state HOTA (SsHOTA) method. These findings confirm the robustness of NsHOTA for real-world wind turbine condition monitoring and highlight its potential integration into predictive maintenance systems.

## 1. Introduction

Electrical machines, and in particular induction machines (IMs), are the cornerstone of a wide range of industrial applications due to their simplicity, robustness, high efficiency, and low cost. Their ubiquity extends from manufacturing systems and transportation to renewable energy conversion in wind turbines (WTs), where they are often implemented as doubly-fed induction generators (DFIGs). With global wind energy capacity expected to reach 2110 GW by 2030 [1], the reliability of these machines becomes increasingly critical to ensure continuous operation. Despite their many advantages, IMs are not immune to failures. Stator winding faults, rotor asymmetries, eccentricities, and bearing defects are among the most common, often leading to severe consequences such as unplanned shutdowns, the loss of productivity, and increased maintenance costs [2]. As industry moves towards more demanding reliability and efficiency targets, minimizing operation and maintenance (O&M) costs while ensuring system availability has become a priority [3,4].

Predictive maintenance has therefore become a fundamental strategy to guarantee the reliable operation of industrial systems. Within this framework, condition monitoring (CM) techniques have attracted considerable attention, as they allow the detection of incipient faults, the monitoring of their progression, and the implementation of timely corrective actions. Among the different methods proposed, motor current signature analysis (MCSA) has emerged as one of the most widespread techniques for fault detection in IMs [5,6]. Its popularity stems from its minimal hardware requirements—current clamps are often sufficient—and its ability to provide valuable diagnostic information without the need for intrusive sensors.

Over the last few decades, the research community has enriched MCSA with advanced signal processing techniques such as the fast Fourier transform (FFT) [7], short-time Fourier transform (STFT) [8], wavelet transform [9,10], and more recently time–frequency representations based on the Hilbert–Huang transform [11], as well as autoregressive-based spectral estimation approaches for improved fault diagnosis under varying operating conditions [12]. These tools have improved the sensitivity and robustness of fault detection. However, traditional FFT-based approaches assume steady-state operation and thus lose accuracy under non-stationary or transient conditions [13]. This limitation is particularly relevant in wind energy applications, where IMs and doubly-fed induction generators (DFIGs) operate under highly variable wind speed profiles, causing fluctuations in load, slip, and voltage. As a result, conventional techniques often fail to reliably diagnose faults under real operational conditions [14].

To overcome these shortcomings, researchers have increasingly investigated more sophisticated approaches. Online CM systems incorporating artificial intelligence (AI) have been proposed, with algorithms based on neural networks, support vector machines (SVMs), or principal component analysis (PCA) [15]. These methods have shown promise for enhancing diagnostic accuracy and enabling early fault detection. Nonetheless, their practical application faces several challenges: the need for large training datasets covering a variety of machines, fault severities, and operating conditions; the difficulty of replicating real-world variability in laboratory test benches; and the high computational resources often required for data processing. Moreover, the interpretation of results from such systems typically demands skilled personnel, which limits their scalability to large fleets of machines.

In parallel, other researchers have focused on time–frequency and order tracking analysis (OTA) methods as an alternative for variable-speed systems. These techniques allow the re-scaling of frequency components into harmonic orders that remain invariant to speed changes, thus improving fault feature extraction in non-steady conditions [16,17,18,19,20]. This family of methods has proven effective in the vibration and current analysis of rotating machinery, including wind turbine DFIGs.

Almost all fault and degradation diagnosis systems depend primarily on two phases: the first phase, obtaining fault features, and the second phase, developing and using an analysis system to interpret these features and deduce the fault or degradation.

Currently, significant progress has been made in the second phase on analysis systems, particularly with the application of artificial intelligence. However, obtaining fault features still has a significant room for improvement. And no matter how well the analysis system analyzes fault features, the precision capable of detecting the fault or degradation will always be limited to the maximum precision intrinsic in the fault features acquired in the first phase.

For this reason, the implementation of the NsHota method has been proposed as an objective to obtain high-precision failure features for any wind turbine operation regime.

Furthermore, the reliable diagnosis of electrical machines under realistic, variable operating conditions remains an open challenge. In wind turbines, this challenge is even more pronounced, since DFIGs—one of the most common generators in variable-speed WTs—are continuously subjected to stochastic wind inputs and grid perturbations. Under these circumstances, diagnostic techniques must not only detect faults under non-stationary conditions but also provide high-precision faulty patterns that can be readily interpreted without requiring advanced expertise.

The present paper applies the Non-steady-state Harmonic Order Tracking Analysis (NsHOTA) technique to address these needs. NsHOTA, previously introduced as an extension of the steady-state HOTA method [21,22], is based on time–frequency analysis and enables the reliable detection and localization of fault components in the current spectrum of DFIGs under both steady and non-steady operating conditions. Unlike conventional methods, NsHOTA produces consistent diagnostic patterns and improves the detection accuracy of each type of fault for each type of fault, simplifying the interpretation of results and facilitating deployment in industrial environments where non-specialized personnel are in charge of maintenance who only need to analyze 15 values. This also enables the creation of comprehensive historical records thanks to the reduced storage requirement, an essential feature in fault diagnosis, where tracking the evolution of a harmonic over time is more indicative than considering its instantaneous value solely.

In this study, NsHOTA is improved and validated using data collected from an in-service WT DFIG, representing one of the few contributions in the literature based on real operational machines rather than laboratory test benches [16,17,18,19,20]. Validation of the method with field data, naturally affected by grid disturbances, converter harmonics, and environmental noise, shows that fault-related harmonics will remain clearly detectable with this approach. NsHOTA tracks their time–frequency trajectories, while noise-induced components lack the required temporal and spectral coherence. Additionally, the improvement in accuracy of this method comes in part from a better time–frequency window adjustment algorithm.

By bridging the gap between theoretical advances and practical implementation, this work contributes to improving the reliability of CM systems in wind energy applications, reducing O&M costs, and ultimately supporting the large-scale integration of wind power in the global energy mix.

The remainder of this paper is organized as follows: Section 2 presents the theoretical framework and implementation of the NsHOTA method. Section 3 discusses the experimental validation on an operating WT. Section 4 highlights the implications and limitations of the approach. Finally, Section 5 concludes this paper and outlines future directions for research.

## 2. The Harmonic Order Tracking Analysis (HOTA) Method

It is well-known that any type of fault induces or amplifies a series of harmonic components in the electric current of an induction machine (IM) [23]. For example, in the case of mixed eccentricity, the frequencies of the fault-related components can be studied in the stator current as Equation (1):

(1) $$f_{ecc}^{s}=f_{s}\pm kf_{r}\;\;\;\;\;\;\;\;\;k=1,2,3,\ldots$$

where $f_{s}$ is the main frequency, *k* is the harmonic order, and $f_{r}$ is the rotor frequency (mechanical rotational speed).

In a DFIG, the frequencies present in the rotor current related to mixed eccentricity are defined as Equation (2):

(2) $$f_{ecc}^{r}=sf_{s}\pm kf_{r}\;\;\;\;\;\;\;\;\;k=1,2,3,\ldots$$

where *s* is the slip.

As can be seen from Equations (1) and (2), the working conditions, particularly the rotational speed, have a major influence on the location of the fault-related components. As a particular feature of WT DFIGs compared to common IMs, these machines can operate both in super- and sub-synchronous regimes, meaning that the slip can take either negative or positive values, respectively. Additionally, conventional frequency-based diagnosis techniques require the signal to be stationary and are therefore limited when applied to DFIGs, which commonly work under non-stationary conditions. To overcome this limitation, several authors have proposed time–frequency domain techniques such as wavelets [24,25]. However, these approaches demand skilled analysts to interpret the time–frequency evolution of harmonic components, which restricts their use in routine maintenance.

To address these issues, the HOTA technique was proposed in [22] and later extended for time-varying conditions in [21]. The method processes the time–frequency information of the rotor current to locate the fault component at the harmonic orders *k*. As a result, a diagram displaying only the information corresponding to the fault at the same position, given by the harmonic orders *k*, irrespective of speed variations, is generated. Maintenance personnel only need to compare these values with theoretical thresholds to identify the presence or absence of a specific fault. These thresholds can be established analytically from the expected fault-related harmonic amplitudes in healthy and faulty states or empirically from baseline data of healthy turbines. Furthermore, temporal analysis of these failure features allows maintenance personnel to study the evolution of the fault degradation.

This approach provides a unique pattern for each type of fault, regardless of the working condition, allowing a reliable diagnostic decision even for non-expert operators. In addition, fault information can be condensed into approximately 15 numerical values (corresponding to the amplitudes of the main harmonic orders and their surroundings for inter fault noisy background analysis), instead of thousands of time–frequency data points. This reduction reduces both storage and transmission requirements in the Condition Monitoring System (CMS).

The present work applies the extended version [21], referred to as NsHOTA. This technique offers specific improvements compared with the original method [22], named SsHOTA. Whereas SsHOTA requires signals acquired under steady-state operation, NsHOTA can process both steady and transient measurements. This capability is particularly relevant for WT diagnosis, since these machines typically operate under variable conditions. In addition, NsHOTA provides higher fault identification precision and lower background noise in all *k*-order spectrum samples. These improvements hold for both steady and non-steady operating regimes.

As follows, operating data gathered from an in-service WT DFIG (see Section 3) are used to explain the NsHOTA procedure, Section 2.1, and the mentioned improvements of NsHOTA compared with SsHOTA, next in Section 2.2.

### 2.1. NsHOTA Steps

**Figure 1:**
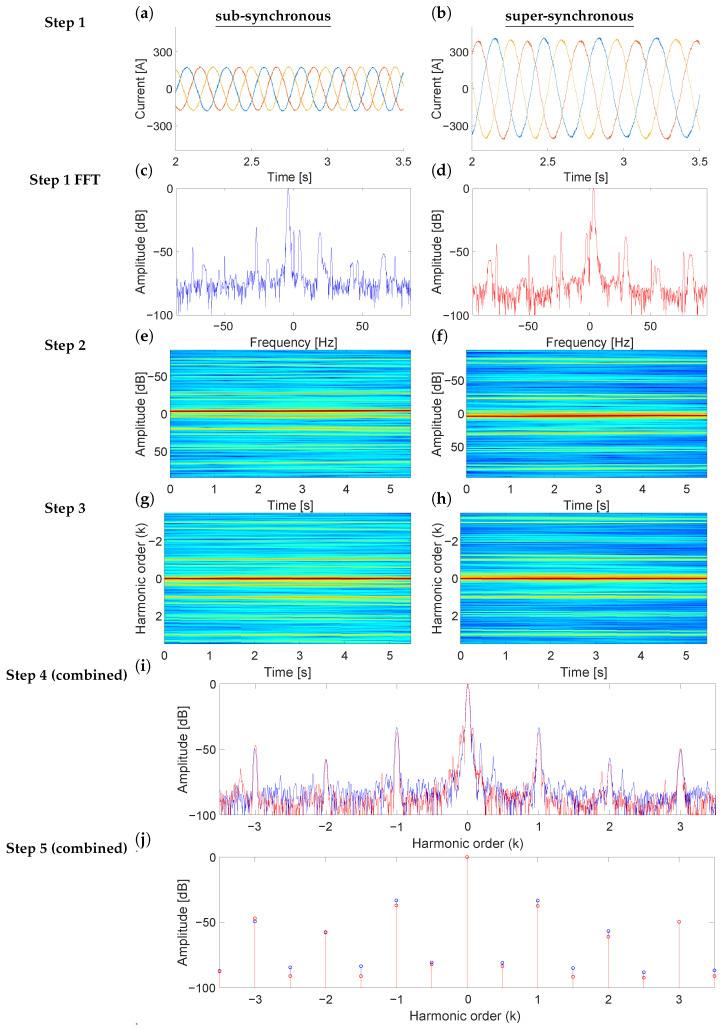
Theoretical explanation of the NsHOTA method. (**a**) Step 1 sub-synchronous current signal [blue: R current, red: S current, yellow: T current], (**b**) Step 1 super-synchronous current signal [blue: R current, red: S current, yellow: T current], (**c**) FFT of sub-synchronous signal, (**d**) FFT of super-synchronous signal, (**e**) Step 2 sub-synchronous time-frequency map [continuous color scale representing amplitude from blue (−100 db) to red (0 db)], (**f**) Step 2 super-synchronous time-frequency map [continuous color scale representing amplitude from blue (−100 db) to red (0 db)], (**g**) Step 3 sub-synchronous harmonic map [continuous color scale representing amplitude from blue (−100 db) to red (0 db)], (**h**) Step 3 super-synchronous harmonic map [continuous color scale representing amplitude from blue (−100 db) to red (0 db)], (**i**) Step 4 combined spectrum [blue: sub-synchronous signal results, red: super-synchronous signal results], and (**j**) Step 5 combined harmonic signature [blue: sub-synchronous signal results, red: super-synchronous signal results].

In this work, the NsHOTA method is applied to the diagnosis of an eccentricity fault. The procedure is summarized below and illustrated in Figure 1 for both super- and sub-synchronous regimes of the in-service WT under study. Thanks to the fact that NsHOTA first compresses the three-phase information through the Park modulus, then obtains a high-resolution time–frequency representation via the Gabor transform, and finally enhances robustness through a weighted median filter, each stage contributes to extracting the fault-related harmonic content with high accuracy and stability.

**Figure 2:**
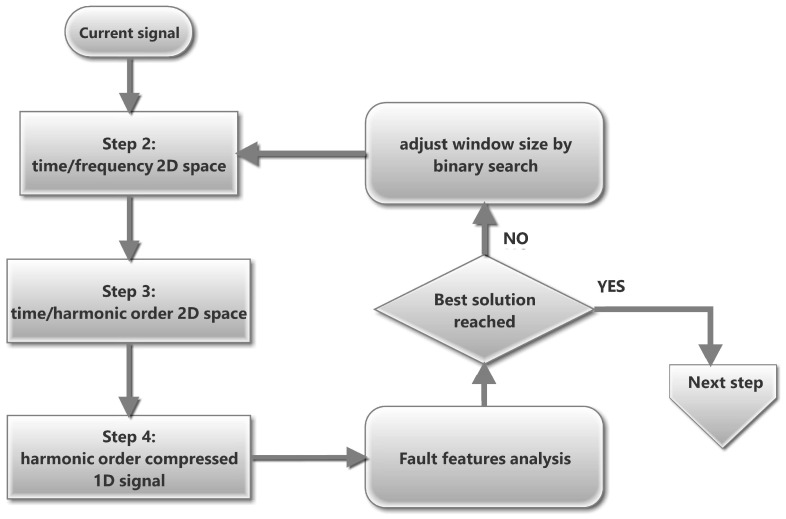
Time/frequency space window adjustment algorithm to obtain high-precision fault features.

1. **Signal acquisition.** The first step consists on acquiring the rotor-side converter currents for each phase (a, b, c). Figure 1a,b shows three -phase rotor current samples acquired with the WT DFIG operating at sub- and super-synchronous regimes, respectively. Figure 1c,d display the corresponding FFT spectra traditionally used for diagnosis. Note that the spectral distribution varies with the operating condition, requiring expert knowledge for interpretation and restricting analysis to steady-state periods.
2. **Park and Gabor transforms.** The three-phase rotor currents are first expressed in their *d*–*q* components ($i_{dr}$ and $i_{qr}$) using Park’s transform as Equations (3) and (4) [26]:
  (3) $$i_{dr}=\sqrt{2/3}\;i_{a}\left(t\right)-\frac{1}{\sqrt{6}}\;\left[i_{b}\left(t\right)+i_{c}\left(t\right)\right]$$
  (4) $$i_{qr}=\frac{1}{\sqrt{2}}\;\left[i_{b}\left(t\right)-i_{c}\left(t\right)\right]$$
  The Park transformation projects the three-phase system into an orthogonal reference frame, removing redundant information and allowing more compact representation of the electrical behavior. Once the Park vector is obtained, the time–frequency analysis is performed using the Gabor transform [27], which provides the energy of the different fault components in the time–frequency plane. The Gabor transform is chosen for its high energy concentration and robustness to noise, enabling the processing of measurements acquired under non-steady conditions. This step is illustrated in Figure 1e,f. The combination of the Gabor transform and the Park transform in NsHOTA is motivated by their complementary roles in signal representation. The Park transform converts the three-phase quantities into a rotating reference frame where the modulus of the Park signal becomes DC (0 Hz). This operation decouples the analysis from the grid frequency and suppresses its possible fluctuations and leakage, ensuring that the fundamental does not interfere with the dynamic components of interest. Applying Gabor transform to the Park-domain signals further concentrates the spectral content around zero frequency, effectively reducing the bandwidth required for accurate representation. This compression allows the hardware front-end and acquisition systems to operate with lower sampling and filtering demands. Finally, performing the FFT on the Gabor-domain signals ensures spectral orthogonality.
3. **Re-scaling the frequency axis.** Once the spectrogram is obtained, the next step consists in transforming the frequency axis so that the fault components appear at their corresponding harmonic order *k*, independently of the operating speed. For an eccentricity fault, the fault-related frequencies are given in Equation (2). To achieve a consistent representation, the time–frequency distribution is re-scaled for each time instant $t_{j}$ according to Equation (5):
  (5) $$f_{j}^{T}=\frac{f_{j}-s_{j}f_{j}^{s}}{f_{j}^{r}}$$
  where $f_{j}^{T}$ is the transformed frequency corresponding to $f_{j}$, and $s_{j}f_{j}^{s}$ represents the main component in the spectrogram at time $t_{j}$, identified as the frequency with the highest energy. The slip $s_{j}$ for each instant $t_{j}$ can be computed from the relationship between stator and rotor frequencies, as shown in Equation (6):
  (6) $$s_{j}=\frac{f_{j}^{s}-f_{j}^{r}}{f_{j}^{s}}\;\;\;\Rightarrow \;\;\;f_{j}^{r}=f_{j}^{s}-s_{j}f_{j}^{s}$$
  In a DFIG connected to the grid, $f_{j}^{s}$ corresponds directly to the network frequency, which eliminates the need for a separate speed measurement. Consequently, the transformed frequency of order *k* can be expressed as Equation (7):
  (7) $$\begin{array}{c} f_{kmecc,j}^{T}=\frac{s_{j}f_{j}^{s}+k\left(f_{j}^{s}-s_{j}f_{j}^{s}\right)-s_{j}f_{j}^{s}}{f_{j}^{s}-s_{j}f_{j}^{s}}=k,\\ \;\;\;\;\;k=\pm 1,\pm 2,\pm 3,\ldots \end{array}$$
  Therefore, in the transformed spectrogram, all fault-related components are aligned at fixed harmonic order positions (*k*), regardless of variations in speed or load. This property allows a direct and consistent comparison of fault features, as illustrated in Figure 1g,h for the sub- and super-synchronous regimes, respectively.
4. **Averaging the harmonic energy**. Since the fault components are aligned at fixed *k*-order positions regardless of the operating condition, their energy distribution can be conveniently condensed into a single representation. This new representation, referred to as the average HOTA spectrum, is analogous to the traditional steady-state spectrum (Figure 1c,d), but significantly easier to interpret. For each transformed frequency, the mean energy is calculated along the entire acquisition period, providing a compact summary of the fault-related information. Performing this compaction of the time-harmonic order space provides another important advantage: Any noise frequencies that are not perfectly aligned with the fault features throughout the time dimension are filtered out. Figure 1i illustrates this step.
5. **Improving the accuracy of fault features.** To obtain a well-defined fault-feature space, this study incorporates an additional refinement based on selecting a window filter with appropriate time- and frequency-domain dimensions. The optimal window depends on the characteristics of the fault harmonics and corresponds to the Gabor window whose parameters are adjusted according to the slopes of the fault components to be detected. The methodology, thoroughly presented in [27], ensures maximum energy concentration in the time–frequency domain, approaching the limits imposed by the Heisenberg uncertainty principle, and minimizes the entropy of the resulting time–frequency representation.
  Previous works established the baseline methodology: first, by adopting consensus window dimensions that produced time–frequency spaces of acceptable quality for fault detection [21], and later by introducing an iterative adjustment procedure based on the minimum-entropy criterion, which improved fault-identification accuracy [22]. These contributions form the stationary reference framework up to this point.
  Building on that foundation, the present study introduces a minor extension to account for transient effects, which were not considered in earlier stationary approaches. In this case, the minimum-entropy condition is explicitly sought by adjusting the window parameter within a ±10% range using a three-point binary search algorithm (Figure 2), reducing computational cost.
6. **Data reduction and transmission.** After obtaining the average HOTA spectrum, the most relevant diagnostic information must be stored or transmitted to the control center for further analysis. To minimize data volume while preserving diagnostic accuracy, HOTA retains only the mean energy values corresponding to the main harmonic orders (*k* from −3 to 3, including the main component) together with selected intermediate points. These approximately 15 values form the harmonic signature of the machine, which allows clear differentiation between fault-related components and background noise. The resulting *k*-Order values, shown in Figure 1j, can be directly compared with predefined thresholds to assess the presence or absence of faults. This compact representation facilitates quick and reliable diagnosis, even for non-specialized personnel.

### 2.2. Improvements in NsHOTA vs. SsHOTA

**Figure 3:**
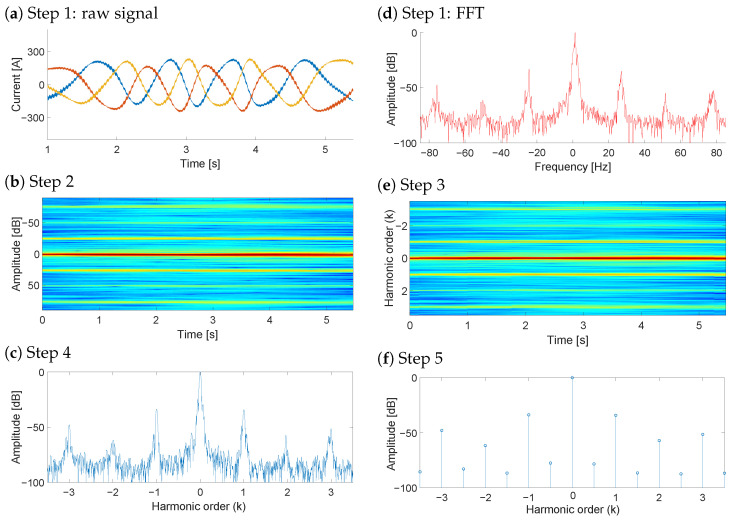
Application of the proposed NsHOTA method to a heavy transient signal from WT DFIG rotor current data. (**a**) Raw signal [blue: R current, red: S current, yellow: T current], (**b**) FFT spectrum [continuous color scale representing amplitude from blue (−100 db) to red (0 db)], (**c**) Step 2 transformation, (**d**) Step 3 analysis, (**e**) Step 4 combined representation [continuous color scale representing amplitude from blue (−100 db) to red (0 db)], and (**f**) Step 5 final harmonic signature.

To ensure a fair and focused evaluation, the proposed NsHOTA method is benchmarked against SsHOTA, as both share the same harmonic-order tracking principle. This design choice isolates the contribution of the non-stationary extension without introducing confounding factors from fundamentally different diagnostic paradigms, which could obscure the specific advantages of NsHOTA. As previously mentioned, the main advantage of the NsHOTA method lies in its capability to process non-steady-state measurements, where are very common during WT operation. Figure 3 illustrates the complete procedure applied step by step to a rotor current signal acquired during a transient period in the sub-synchronous regime.

**Figure 4:**
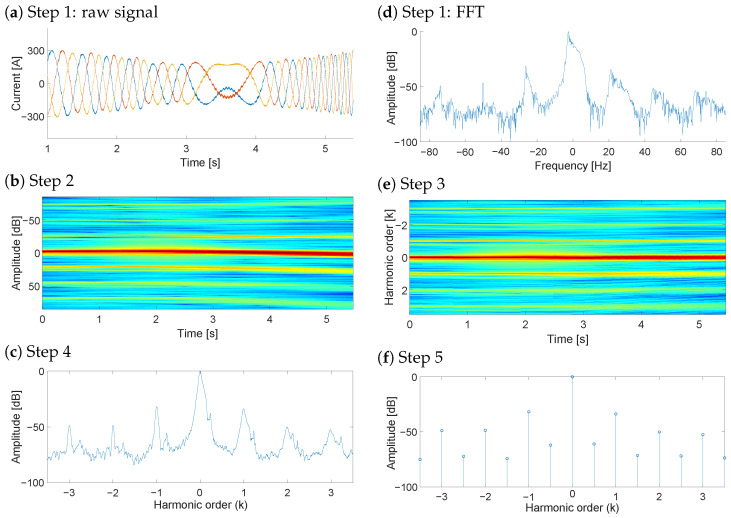
Application of the proposed NsHOTA method to WT DFIG rotor current data acquired during a transient condition. (**a**) Raw signal [blue: R current, red: S current, yellow: T current], (**b**) FFT spectrum [continuous color scale representing amplitude from blue (−100 db) to red (0 db)], (**c**) Step 2 transformation, (**d**) Step 3 analysis, (**e**) Step 4 combined representation [continuous color scale representing amplitude from blue (−100 db) to red (0 db)], and (**f**) Step 5 final harmonic signature.

A second example of a transient signal analyzed with NsHOTA is presented in Figure 4. In this case, the WT DFIG transitions from the sub-synchronous to the super-synchronous operation. Even under these rapidly changing conditions, the NsHOTA method successfully extracts the fault-related information, demonstrating its robustness to dynamic operating regimes.

**Figure 5:**
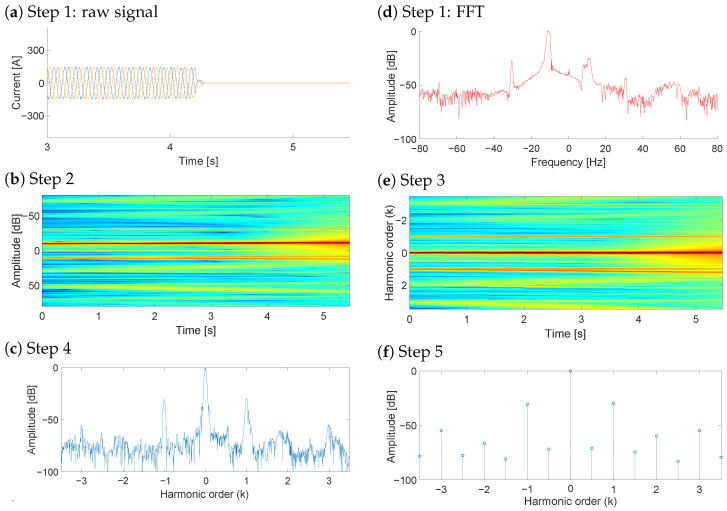
Fault features obtained from a non-steady WT DFIG rotor current signal with an abrupt end, processed using the proposed NsHOTA technique. (**a**) Raw signal [blue: R current, red: S current, yellow: T current], (**b**) FFT spectrum [continuous color scale representing amplitude from blue (−100 db) until red (0 db)], (**c**) Step 2 transformation, (**d**) Step 3 analysis, (**e**) Step 4 combined representation [continuous color scale representing amplitude from blue (−100 db) until red (0 db)], and (**f**) Step 5 final harmonic signature.

Another important improvement concerns the ability of NsHOTA to recover information from partially degraded or incomplete signals. The so-called fault energy maximization algorithm minimizes the influence of poor-quality data segments, preserving the key fault signatures. Figure 5 shows an example in which the rotor current suddenly drops to zero, probably due to a temporary decrease in wind speed below the cut-in threshold. Despite this disturbance, the NsHOTA algorithm is able to reconstruct the characteristic fault features effectively.

**Figure 6:**
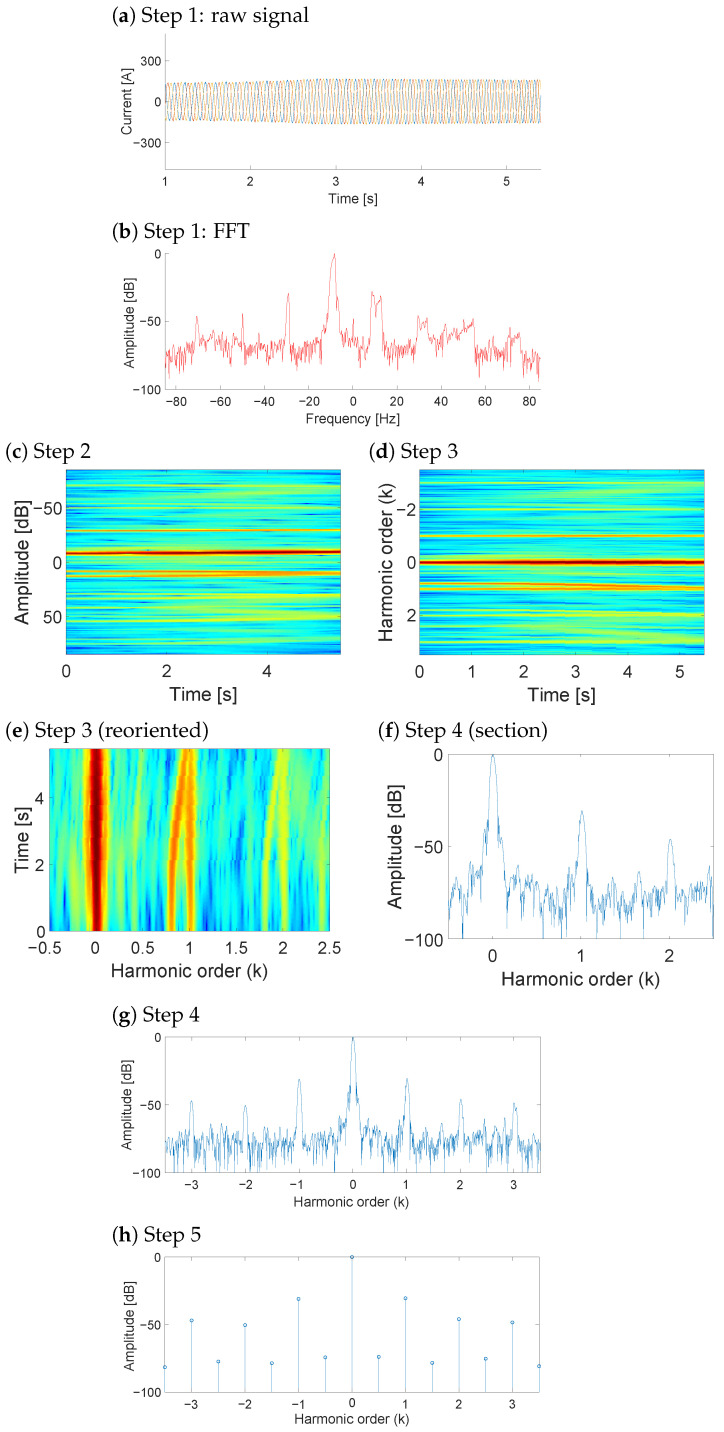
Fault features obtained from a non-steady WT DFIG rotor current signal with interference frequency filtered, processed using the proposed NsHOTA technique. (**a**) Raw signal [blue: R current, red: S current, yellow: T current], (**b**) FFT spectrum, (**c**) Step 2 transformation [continuous color scale representing amplitude from blue (−100 db) to red (0 db)], (**d**) Step 3 analysis [continuous color scale representing amplitude from blue (−100 db) to red (0 db)], (**e**) Step 3 reoriented view [continuous color scale representing amplitude from blue (−100 db) to red (0 db)], (**f**) Step 4 section, (**g**) Step 4 combined representation, and (**h**) Step 5 final harmonic signature.

Furthermore, NsHOTA incorporates a weighted-median filtering process that converts the *k*-Order map into a compact *k*-Order vector. An example of this enhancement is shown in Figure 6. Through this conversion, frequency components that do not evolve in parallel with the true fault trajectories in the time/*k*-Order space are filtered out. Consequently, compared with the original SsHOTA method, NsHOTA provides a cleaner spectrum with reduced noise and minimal overlap between fault components.

Figure 6e presents a reoriented section of the time/*k*-Order plane, covering harmonic orders from −0.5 to 2.5, where the axes have been switched with respect to Step 3 (Figure 6d). This reorientation allows visualizing the temporal evolution of the amplitudes, a feature that was not available in SsHOTA. Since the main frequency and fault-related frequencies in NsHOTA appear almost parallel to the *y*-axis, all amplitudes deviating from this alignment can be efficiently suppressed using the weighted median. The effect of this filtering is illustrated in Figure 6f, where the amplitudes close to but not perfectly parallel to the fault trajectories are successfully removed, improving the accuracy in detecting the true fault-related frequency components. Fault-related harmonics remain identifiable even in noisy environments, since NsHOTA tracks them as time–frequency trajectories rather than fixed frequency peaks. Noise and grid effects may introduce additional harmonics, but these are not coherent over time and therefore do not follow the characteristic evolution of fault components [28,29]. Consequently, the algorithm isolates only those harmonics physically linked to specific degradations, which increases robustness against spectral interference compared to conventional steady-state methods. In summary, NsHOTA enhances the robustness and interpretability of current-based fault diagnosis in WT DFIGs. Its ability to handle transient signals, recover useful data from degraded measurements, and suppress noise through weighted-median filtering results in more precise fault identification and a cleaner harmonic representation than that obtained with the traditional SsHOTA approach.

## 3. In-Service WT Data Used for the Analysis and Background

**Figure 7:**
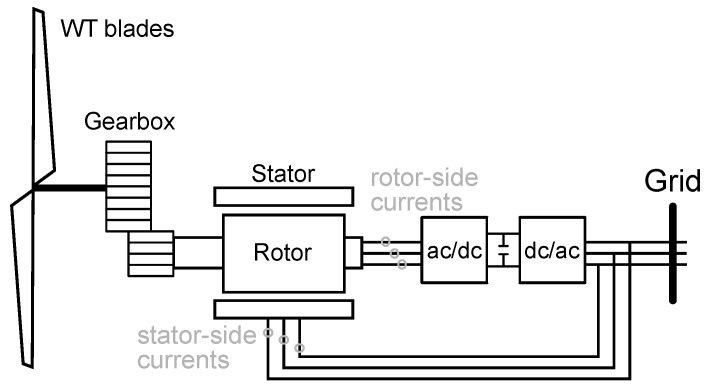
DFIG Diagram with location of current sensors.

The WT under analysis is an 850 kW, two-pole-pair doubly-fed induction generator (DFIG) that was previously diagnosed with mixed eccentricity [30]. The dataset used in this study consists of three-phase rotor and stator current signals recorded continuously over an eight-month period of WT operation. The location of the current sensors installed in the WT DFIG is shown in Figure 7, while the types of current measurements employed for the analysis are summarized in Table 1.

A preliminary analysis of the same DFIG was conducted in [31] using the steady-state version of the Harmonic Order Tracking Analysis (SsHOTA) method. As described in Section 2, the SsHOTA technique requires stationary operating conditions to ensure an accurate frequency-based diagnosis. Therefore, before applying SsHOTA, each recorded signal had to be classified as stationary or non-stationary, and only the former were used. The criteria employed to discriminate between permanent and transient regimes are detailed below. Each measurement was divided into eight consecutive segments, and for each segment and each of the three phases, the following parameters were computed:

- RMS value of raw stator currents.
- Mains frequency of stator currents.
- RMS value of raw rotor-side converter currents.
- Mains frequency of rotor-side converter currents.

A measurement was classified as stationary when the mains frequencies of the stator and rotor-side currents remained constant and the difference between their RMS values was below 10%. Table 2 summarizes the number of measurements meeting these stationary criteria and the total number of available measurements per month.

In the previous study [31], only 1285 stationary signals were suitable for analysis with the SsHOTA method. In contrast, the present work employs all 3861 available signals by using the non-steady-state version of the method (NsHOTA). This capability represents a major advantage, as it eliminates the need to preselect signals or implement the aforementioned discrimination algorithm. Furthermore, the inclusion of all available measurements increases the amount of information available for analysis, simplifies fault localization, and significantly enhances diagnostic reliability and robustness.

## 4. Results: Application of HOTA to Steady and Non-Steady State Working Conditions

**Figure 8:**
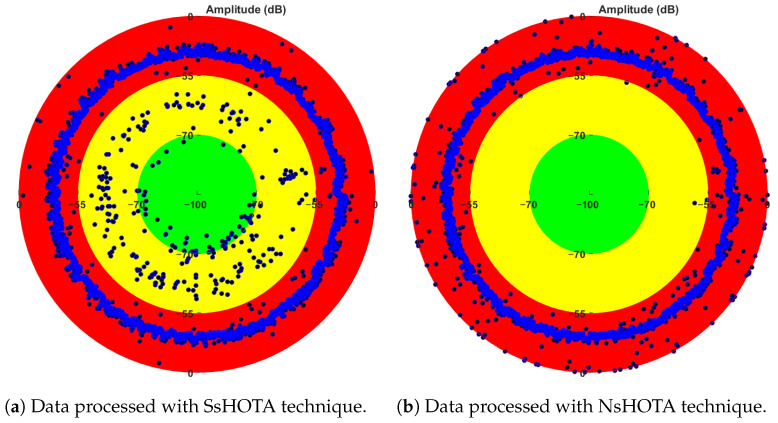
Polar plot of fault feature distribution obtained from eight months of WT DFIG data. Green: no fault; yellow: incipient fault; red: confirmed fault.

The results obtained by applying both the SsHOTA and NsHOTA methods to all available measurements (stationary and non-stationary) from the eight-month WT DFIG dataset described in Section 3 are presented in Figure 8. The results are displayed using a polar plot representation, where each point corresponds to a measurement and the data are ordered chronologically. Three color-coded zones indicate the fault condition: green represents healthy operation (no fault detected), yellow corresponds to an incipient fault, and red indicates the confirmed presence of a fault.

As illustrated in Figure 8, the difference in performance between the two approaches is evident. When applying the SsHOTA method (Figure 8a), a considerable number of samples are misclassified, resulting in a wide dispersion across the green and yellow zones. This behavior reflects the inherent limitations of SsHOTA when processing non-stationary signals. In contrast, the NsHOTA method (Figure 8b) successfully classifies the entire set of WT DFIG fault features, with all samples consistently falling within the red zone. This improvement stems from the generation of the time/*k*-Order space, which enables a more detailed representation of fault-related components and a more accurate analysis of dynamic behavior.

The enhanced performance of NsHOTA is also attributed to the implementation of the *fault energy maximization algorithm*, which adaptively selects the most suitable size of the RBF window used to construct the *k*-Order/time space. This adaptive process minimizes the occurrence of false negatives by mitigating the influence of abnormal or low-quality signal segments. Consequently, the NsHOTA method demonstrates strong robustness against false fault suppression under non-steady-state operating conditions.

**Figure 9:**
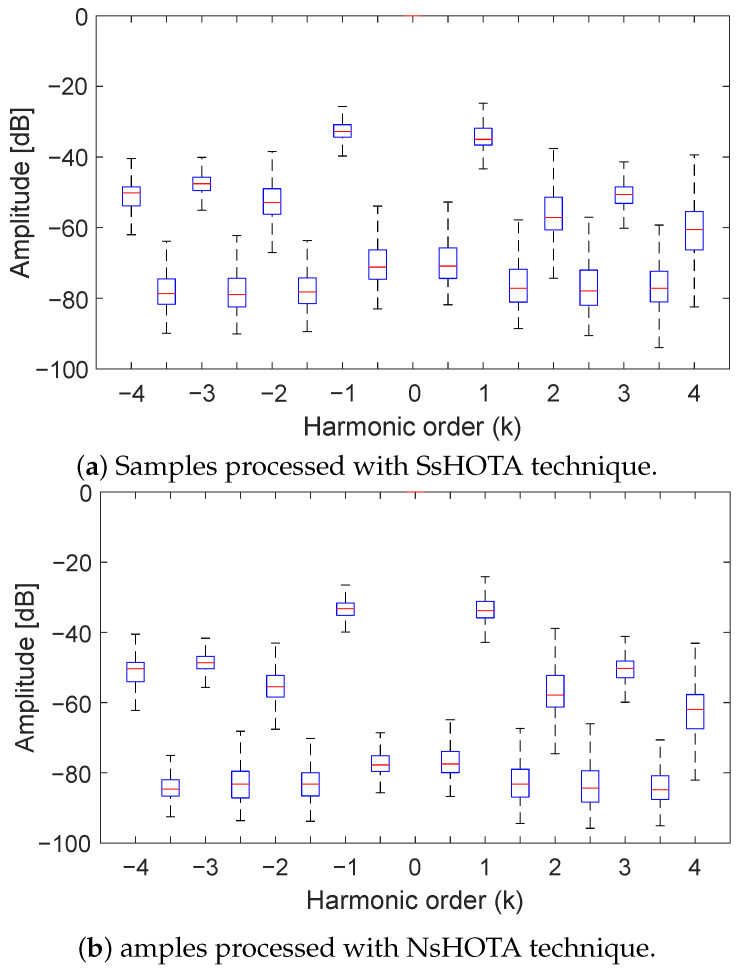
Comparison of fault-feature dispersion from eight months of WT DFIG data using (**a**) SsHOTA and (**b**) NsHOTA. The narrower boxes in (**b**) reflect the higher diagnostic precision of NsHOTA.

To further quantify these improvements, Figure 9a,b present box-and-whisker plots comparing the results obtained with SsHOTA and NsHOTA, respectively. The *k*-integer values (excluding k=0) represent the fault-related harmonic orders expressed in decibels, while the intermediate values correspond to the mean noise background level. These parameters are essential for assessing signal quality and interference, as described in Section 2. When comparing both plots, the NsHOTA results (Figure 9b) clearly exhibit reduced percentile deviation and lower background noise, confirming a substantial improvement in diagnostic precision. This increased precision in fault-feature extraction is crucial for accurate long-term monitoring and degradation assessment of WT DFIGs.

It is important to note that the NsHOTA approach is not mutually exclusive with other adaptive time–frequency techniques such as synchrosqueezing. Synchrosqueezing could be applied after the Gabor transform used in NsHOTA to adjust for the frequency variations generated by operating in a transient regime. The subsequent harmonic-order mapping performed by NsHOTA would then benefit from this enhanced representation. Thus, NsHOTA can be combined with advanced time–frequency techniques to refine diagnostic accuracy further. Consequently, NsHOTA can be integrated with such time–frequency refinement techniques to further increase robustness and sensitivity in real-world applications.

## 5. Conclusions

Wind turbines (WTs) are becoming increasingly complex as their rated power increases and as both mechanical and electrical subsystems evolve toward higher efficiency and control precision. This increased complexity, however, results in more demanding maintenance procedures and higher failure-related costs. To minimize downtime and operational expenses, recent research efforts have focused on predictive maintenance strategies that provide early warnings of potential failures. Nevertheless, achieving high-precision fault detection together with clear and interpretable health assessments remains a significant challenge.

Recognizing that effective maintenance strategies can directly reduce the levelized cost of energy (LCOE), this work has proposed a novel diagnostic method for doubly-fed induction generators (DFIGs) in wind turbines. The proposed Non-steady-state Harmonic Order Tracking Analysis (NsHOTA) technique, based on time–frequency analysis, enables accurate signal processing and feature extraction under both steady and non-steady-state operating conditions. A key advantage of NsHOTA is its ability to map fault-related information to fixed harmonic order positions, producing a distinctive and invariant pattern for each fault type. This property facilitates straightforward interpretation of diagnostic results, even by non-specialized personnel, and ensures robustness across variable operating conditions. Furthermore, NsHOTA significantly reduces the volume of data by condensing fault-related information into a compact set of representative values, in contrast to the thousands of points typically required by traditional frequency or time–frequency approaches.

For the first time, the NsHOTA method has been implemented and validated using eight months of field data collected from an in-service WT DFIG affected by an eccentricity fault. Because the method can process both steady and transient conditions, all available measurements were analyzed without the need for pre-filtering. This capability leads to a simpler, faster, and more reliable diagnostic process. Moreover, NsHOTA can detect variations in the amplitude of fault components and classify fault severity, enabling early-stage detection and continuous tracking of fault progression throughout the turbine’s operational life.

This study demonstrated the robustness of NsHOTA for fault diagnosis in an in-service DFIG wind turbine operating under non-stationary conditions, leveraging long-term field data rather than laboratory experiments. The proposed approach effectively captures harmonic-order dynamics and provides reliable diagnostic insights in realistic environments. Additionally, exploring hybrid strategies that combine NsHOTA with advanced time–frequency preprocessing or autoregressive-based spectral estimation could further enhance diagnostic accuracy and adaptability in complex operational scenarios. We also plan to evaluate NsHOTA across different fault types, power ratings, and compound or progressive faults to demonstrate its general applicability. The current work establishes the proof of concept and field validation necessary to support these broader studies.

## Figures and Tables

**Table 1:** Characteristics of the signals used for the analysis.

Current Transducer	Sampling Parameters	Measurement Type
HOP 2000-SB/SP1 ±3000 A	1.5 kHz 5.4 s	Stator-side current phase *a*
		Stator-side current phase *b*
		Stator-side current phase *c*
		Rotor-side converter current phase *a*
		Rotor-side converter current phase *b*
		Rotor-side converter current phase *c*

**Table 2:** Monthly breakdown of measurements for the operating WT data.

Month	No. of Stationary Measurements	Total Number of Measurements
November	212	528
December	82	281
January	259	693
February	164	519
March	180	652
April	96	382
May	160	433
June	132	373
TOTAL	1285	3861

## Data Availability

The original contributions presented in this study are included in the article. Further inquiries can be directed to the corresponding author.
